# A Robust and Accurate Two-Step Auto-Labeling Conditional Iterative Closest Points (TACICP) Algorithm for Three-Dimensional Multi-Modal Carotid Image Registration

**DOI:** 10.1371/journal.pone.0148783

**Published:** 2016-02-16

**Authors:** Hengkai Guo, Guijin Wang, Lingyun Huang, Yuxin Hu, Chun Yuan, Rui Li, Xihai Zhao

**Affiliations:** 1 Research Institute of Image and Information, Department of Electrical Engineering, Tsinghua University, Beijing, China; 2 Center for Biomedical Imaging Research, Department of Biomedical Engineering, Tsinghua University, Beijing, China; 3 Department of Radiology, University of Washington, 850 Republican St, Seattle, WA, United States of America; 4 Healthcare Department, Philips Research China, Shanghai, China; Shenzhen Institutes of Advanced Technology, CHINA

## Abstract

Atherosclerosis is among the leading causes of death and disability. Combining information from multi-modal vascular images is an effective and efficient way to diagnose and monitor atherosclerosis, in which image registration is a key technique. In this paper a feature-based registration algorithm, Two-step Auto-labeling Conditional Iterative Closed Points (TACICP) algorithm, is proposed to align three-dimensional carotid image datasets from ultrasound (US) and magnetic resonance (MR). Based on 2D segmented contours, a coarse-to-fine strategy is employed with two steps: rigid initialization step and non-rigid refinement step. Conditional Iterative Closest Points (CICP) algorithm is given in rigid initialization step to obtain the robust rigid transformation and label configurations. Then the labels and CICP algorithm with non-rigid thin-plate-spline (TPS) transformation model is introduced to solve non-rigid carotid deformation between different body positions. The results demonstrate that proposed TACICP algorithm has achieved an average registration error of less than 0.2mm with no failure case, which is superior to the state-of-the-art feature-based methods.

## Introduction

Atherosclerotic plaque is prevalent in carotid bifurcation, which is one of the major causes of ischemic stroke [1]. Several non-invasive medical imaging modalities are potential to evaluate the vulnerability of carotid plaque by its morphology and composition [2]. Ultrasound (US) provides a low-cost and real-time method for plaque imaging [3], while magnetic resonance (MR) imaging provides more comprehensive plaque characterization [4] compared with US. It is beneficial to validate US findings with MR to improve the efficiency of vulnerable plaque diagnosis and optimize the turning point of clinical procedure [5]. Thus, it is critical to perform image registration [6] aligning multi-modal image sets from the same patient during image analysis.

Due to huge modality variance and very few accurate anatomical landmarks in carotid artery images [7], multi-modal carotid image registration between MR and US is a challenging task. Several algorithms have been proposed, which can be divided into three categories: feature-based methods [8], intensity-based methods [9][10], and hybrid model methods [11]. *Feature-based methods* first extract landmarks such as vessel centerlines or lumen surfaces from both images. Transformation models among corresponding landmarks are then estimated using point set registration algorithms such as iterative closest points (ICP) [12]. Chui et al. [8] developed a feature-based 3D rigid registration method using ICP algorithm. Optimized rotation matrix was calculated for 3D surfaces after manual lumen contour segmentation and carotid bifurcation alignment for MR and US images. Their methods achieved an average error of less than 1mm among three patients. *Intensity-based methods*, on the other hand, match the image intensity directly using metrics like mutual information(MI) [9][13][10] without extracting features. Furthermore, *hybrid model methods* combine both methods to achieve better registration results. Carvalho et al. [11] introduced a hybrid model into the multi-modal carotid image registration. They combined feature-based and intensity-based algorithms into a cost function, and obtained an average Dice similarity coefficient (DSC) of 0.69 ± 0.08 and mean surface distance (MSD) of 0.87 ± 0.25mm in test set.

Compared with other algorithms, feature-based methods are fast to compute and have a bigger capture range than intensity-based methods [14]. They can serve as independent registration algorithms [8], or good initialization for intensity-based methods to avoid local minimum (landmark matching in [10] and rigid centerline registration in [11]). Moreover, feature-based registration can also form a penalty term in hybrid model methods [11] to constrain the transformation.

Although a variety of feature-based algorithms existed, they all have some limitations: (1) None of current feature-based algorithms for multi-modal carotid image registration employ non-rigid models, which are necessary because of twisting and bending transformation caused by different patient positions during US and MR scan [10]. (2) The best average registration error is 0.55 ± 0.29*mm* with manual adjustment in [8], which is relative large compared to the vessel wall thickness. Such large error may deteriorates the component analysis after registration. (3) Manual alignment of bifurcation is required in [8]. It increases the burden of operators and may introduce extra manual errors for registration.

In this paper, we propose a novel feature-based non-rigid algorithm for multi-modal carotid image registration, Two-step Auto-labeling Conditional Iterative Closest Points (TACICP) algorithm, which takes advantage of label information of the vessels. The label of each point indicates the vessel it belongs to and is assigned automatically. A coarse-to-fine strategy is applied for robustness and accuracy, including rigid initialization step and non-rigid refinement step. Compared with other coarse-to-fine methods in non-rigid registration [15], different calculated features from 2D contours are exploited in two steps for our method. In rigid step, we present conditional iterative closest points (CICP) algorithm for robust initialization and automatic labeling using centerlines. Then in non-rigid step, the CICP algorithm combined with thin-plate-spline (TPS) model is employed for accurate non-rigid refinement with interpolated surface features. Proposed TACICP algorithm is evaluated on our US-MR datasets with three metrics, which reflect robustness, global and local accuracy of registration respectively. Compared with state-of-the-art feature-based algorithms, the TACICP algorithm achieves the best performance under all metrics, whose registration error is less than half of the best results from other methods.

Our contributions are threefold: (1) We present a novel two-step non-rigid registration algorithm between 3D US and MR carotid images, which outperforms the state-of-the-art feature-based methods on US-MR datasets. (2) In TACICP algorithm, we propose an automatic labeling method for vessel categorization to achieve better point set matching accuracy. (3) We design a novel strategy to exploit different models and different features for different steps, which guarantees the robustness and accuracy of the algorithm.

## Methods

### Overview of registration framework

**Fig 1:**
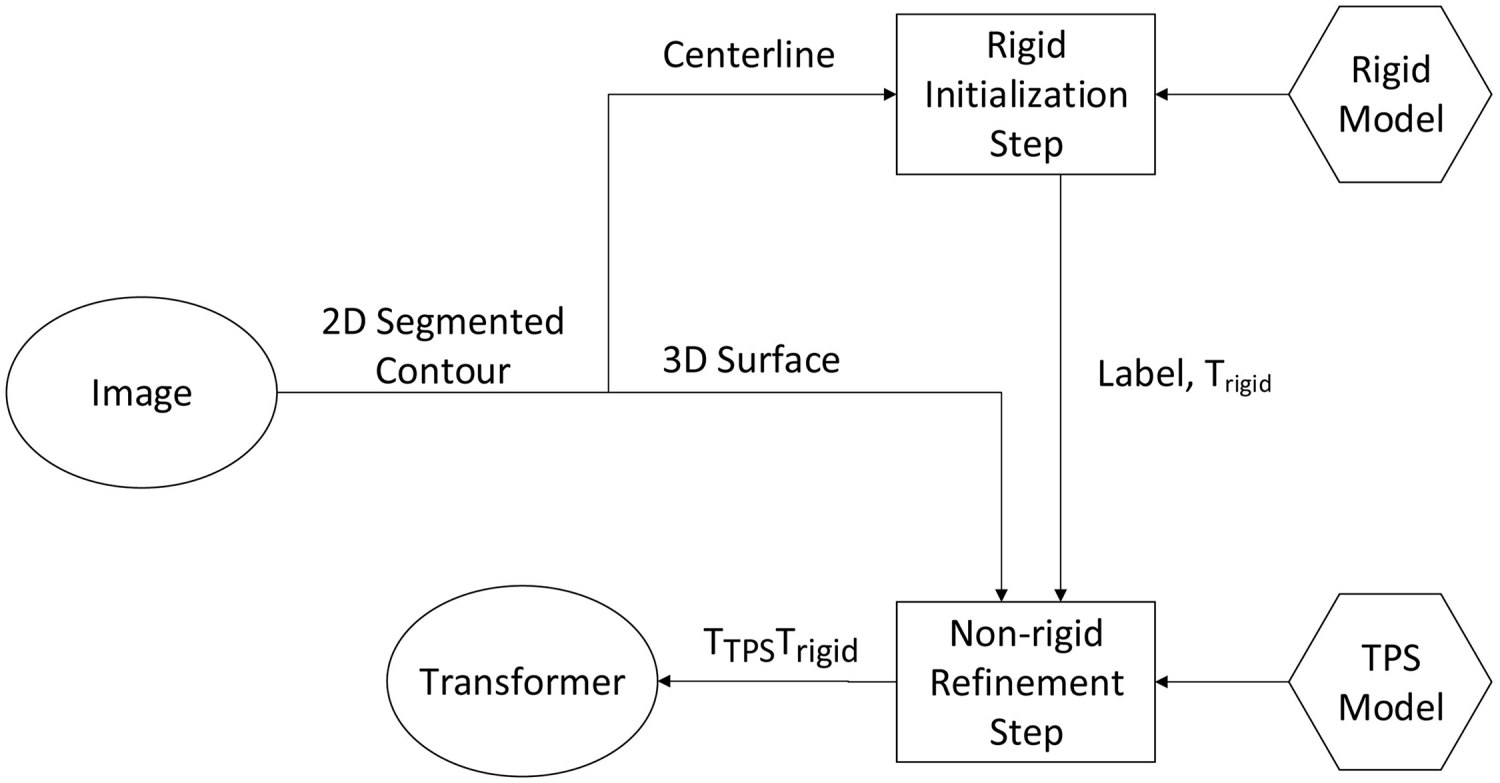
Overview of proposed TACICP algorithm for carotid image registration. The segmented 2D contours from images are the only inputs for our algorithm. Centerline and surface features are generated automatically from contours for two steps. The final output of the registration is a transformation composed by the rigid transformation *T*_rigid_ from rigid initialization step and the thin-plate-spline transformation *T*_TPS_ from non-rigid refinement step.

Shown in Fig 1, the TACICP algorithm consists of two steps: *rigid initialization step* and *non-rigid refinement step*. Each step employs 2D segmented contours from cross-sectional slices as input. An auto-labeling CICP algorithm is employed in rigid initialization step using calculated centerline from contours to obtain robust rigid transformation. Then labels and the same 2D contours after rigid registration are fed into non-rigid step, and a TPS model with surface features reconstructed from contours is optimized to solve the non-rigid deformation caused by different body positions. Final transformation is applied on the images. Next two subsections provide these two steps respectively. The last subsection describes the details of segmentation.

### Rigid initialization step

A feature-based registration algorithm is designed to calculate 3 translation parameters and 3 rotation parameters of rigid transformation as the initialization for non-rigid step. Centerlines are used as registration feature because they contain less noise with average of contour points. They are extracted using the geometrical centroid of 2D contours in each slice. Suppose the 2D contours are made up of line segments between N vertices (*x*_*i*_, *y*_*i*_), *i* = 0, …, *N* − 1, and let (*x*_*N*_, *y*_*N*_) = (*x*_0_, *y*_0_), the centroid (*x*_*c*_, *y*_*c*_) can be formulated as [16]:

$$\begin{array}{ccc} x_{c} & = & \frac{\sum_{i=0}^{N-1}\left(x_{i}+x_{i+1}\right)\left(x_{i}y_{i+1}-x_{i+1}y_{i}\right)}{3\sum_{i=0}^{N-1}\left(x_{i}y_{i+1}-x_{i+1}y_{i}\right)} \end{array}$$ (1)

$$\begin{array}{ccc} y_{c} & = & \frac{\sum_{i=0}^{N-1}\left(y_{i}+y_{i+1}\right)\left(x_{i}y_{i+1}-x_{i+1}y_{i}\right)}{3\sum_{i=0}^{N-1}\left(x_{i}y_{i+1}-x_{i+1}y_{i}\right)} \end{array}$$ (2)

The centroid sets are then interpolated with B-spline and sampled in the interval of 1mm.

ICP algorithm [12] is a classical and effective feature-based registration algorithm. When applying ICP directly on centerline registration, the centerline of internal carotid artery (ICA) and external carotid artery (ECA) may be incorrectly paired. To deal with such local minimum, we introduce vessel category labels as new registration cues. With labels, we propose a new version of ICP algorithm, named *Conditional Iterative Closest Points (CICP)*.

#### Labels and Automatic labeling strategy

Each point **p** on the contours is labeled *L*_*p*_ by its vessel category. Though the reviewers can label them during manual segmentation, sometimes the contours of ECA and ICA in the same slice are difficult to distinguish, especially in US images. To automatically obtain correct label configuration for 2D contours, we employ a labeling strategy for the special bifurcation structure of carotid vessel.

For unlabeled 2D contours, we set the labels of points to common carotid artery (CCA) in all the slices that contain only one contour. For slices with two contours, the label orders are ambiguous: ECA-ICA or ICA-ECA. Notice that the structure of carotid bifurcation are asymmetrical, the wrong configuration will lead to a larger registration error than correct configuration. Our algorithm fixes the labels in fixed images as ECA-ICA and assigns two possible configurations (i.e. ECA-ICA and ICA-ECA) in moving images. The registration errors for both configurations are calculated and the configuration with smaller registration error is automatically chosen as the correct labels. Here we employ LMSD metric as registration error, which will be introduced latter (see Evaluation measures).

#### Conditional Iterative Closest Points (CICP)

Let $M={\left\{m_{i}\right\}}_{i=1}^{N_{m}}$ and $F={\left\{f_{i}\right\}}_{i=1}^{N_{f}}$ from $R^{3}$ be two point sets for registration, denoted by *moving point set* and *fixed point set* with total point number *N*_*m*_ and *N*_*f*_ respectively. The goal of registration is to find the optimal transformation *T* aligning the moving point set to the fixed one. Original ICP algorithm consists of two stages: match stage and transform stage. The match stage aims to find a subset $F^{\prime }={\left\{f_{i}^{\prime }\right\}}_{i=1}^{N_{m}}\subset F$, where $f_{i}^{\prime }$ satisfies:

$$\begin{array}{ccc} f_{i}^{\prime } & = & \underset{f_{j}\in F}{argmin}{∥f_{j}-m_{i}^{\prime }∥}_{2} \end{array}$$ (3)

$$\begin{array}{ccc} & = & \underset{f_{j}\in F}{argmin}d\left(f_{j},m_{i}^{\prime }\right)\;i=1,2,\cdots ,N_{m} \end{array}$$ (4)

where *d*(*p*_1_, *p*_2_) is the Euclidean distance between point *p*_1_ and *p*_2_ and $m_{i}^{\prime }$ is the moving points after applying the estimated transformations in the previous stages. The transform stage is to find an optimal transformation $T_{opt}$ in the model parameter space to minimize the total error:

$$T_{opt}=\underset{T\in T}{argmin}\sum_{i=1}^{N_{m}}{∥f_{i}^{\prime }-T\left(m_{i}^{\prime }\right)∥}_{2}$$ (5)

where T is the parameter space for all possible transformation.

To apply the labels into the match stage, we introduce a symmetric *conditional weight matrix* **W** = (*w*_*i*, *j*_)_3 × 3_, where *w*_*i*, *j*_ is defined as the conditional distance weight between label *i* and label *j*:

$$W=\left(\begin{array}{ccc} w_{0} & w_{1} & w_{1}\\ w_{1} & w_{0} & w_{2}\\ w_{1} & w_{2} & w_{0} \end{array}\right)$$ (6)

where *w*_1_, *w*_2_ ≥ *w*_0_ > 0. By modifying the distance into conditional distance, Eq (3) changes to:

$$f_{i}^{\prime }=\underset{f_{j}\in F}{argmin}d\left(f_{j},m_{i}^{\prime }|L_{f_{j}},L_{m_{i}^{\prime }}\right)\;i=1,2,\cdots ,N_{m}$$ (7)

where *d*(*p*_1_, *p*_2_|*L*_1_, *L*_2_) = *w*_*L*_1_, *L*_2__ *d*(*p*_1_, *p*_2_) is the conditional distance between point *p*_1_ with label *L*_1_ and *p*_2_ with label *L*_2_. We simply set *w*_0_ to 1. Because the matches between ICA and ECA are meaningless, the distance weight *w*_2_ is set to infinity. *w*_1_ is set to 1 due to the ambiguous categorizing between CCA and other vessels near bifurcation. Such setting also guarantees the convergence of CICP algorithm (See the Appendix for details).

**Fig 2:**
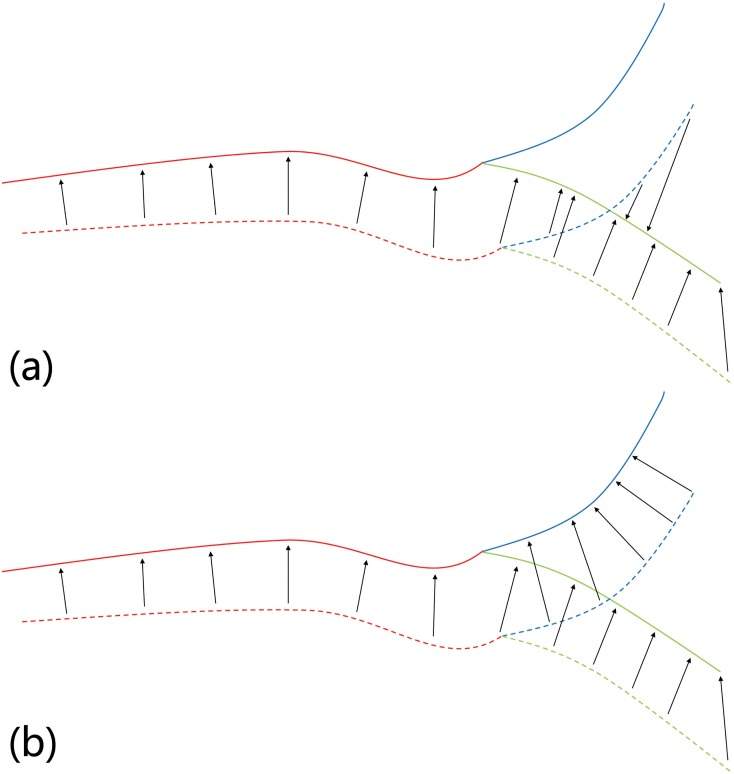
Example of (a) match stage and (b) conditional match stage with the same point set pair at the same position. The same color indicates the same category of points. The solid line represents the fixed point set, and the dash line represents the moving point set. The arrows are partial matching between two point sets.

Fig 2 shows an example of the difference between the match stage in ICP and the conditional match stage in CICP. In fact, ICP is a special version of CICP where all the elements in the weight matrix **W** are set to 1. After using labels in the match stage, the information of vessel categories is added into match stage to promote vessel matching. So the mismatching between vessel categories can be prevented. It is noteworthy that CICP is different from weighted ICP [17]. In CICP algorithm the weights are applied on the match stage, while in weighted ICP algorithms the weights are applied on the transform stage. CICP is also different from three independent regular ICP even when *w*_1_ is set to infinity, because in the transform stage the optimal transformation is computed by considering all the matching pairs with different labels. The independence is only in the match stage, which relies on their labels.

### Non-rigid refinement step

Only rigid model is not enough for multi-modal carotid image registration, since the neck of the patient is in different state of bending and twisting during MR and US imaging. Based on rigid initialization step, a non-rigid transformation is optimized to obtain high registration accuracy. Surfaces interpolated by 3D B-spline from 2D contours are used as features instead of centerline due to more local information in surfaces.

In non-rigid model, registration accuracy is highly dependent on the point set matching, because the model is more flexible than rigid model. To better handle the mismatching between ECA and ICA especially near bifurcation, we employ the same CICP algorithm with the correct label configuration from the first step.

A widely-used model, 3D thin-plate-spline (TPS) model [13], is employed as the non-rigid model in CICP algorithm. It fits a mapping function *f*(**m**_*i*_) between moving point set {**m**_*i*_} and corresponding fixed subset $\left\{f_{i}^{\prime }\right\}$ by minimizing the energy function below:

$$\begin{array}{ccc} & & E_{\text{TPS}}\left(f\right)=\sum_{i=1}^{N_{m}}{∥f_{i}^{\prime }-f\left(m_{i}\right)∥}_{2}^{2}\\ & & +\lambda \;\int \int \int \;\{{\left(\frac{\partial^{2}f}{\partial x^{2}}\right)}^{2}+{\left(\frac{\partial^{2}f}{\partial y^{2}}\right)}^{2}+{\left(\frac{\partial^{2}f}{\partial z^{2}}\right)}^{2}\\ & & +2\left[{\left(\frac{\partial^{2}f}{\partial x\partial y}\right)}^{2}+{\left(\frac{\partial^{2}f}{\partial x\partial z}\right)}^{2}+{\left(\frac{\partial^{2}f}{\partial y\partial z}\right)}^{2}\right]\}dxdydz \end{array}$$ (8)

where *λ* ≥ 0 is a regularization constant. Suppose each point *p* is represented in homogeneous coordinates, i.e. *p* = [1, *p*_*x*_, *p*_*y*_, *p*_*z*_]^*T*^, the mapping function is as follows:

$$f\left(m_{i}\right)=D\cdot m_{i}+P\cdot \phi \left(m_{i}\right)$$ (9)

where **D** is a 4 × 4 matrix for the affine transformation, and **P** is a 4 × *N*_*m*_ warping coefficient matrix for non-rigid deformation. The *υ*(**m**_*i*_) is an *N*_*m*_ × 1 vector for each point **m**_*i*_, where each element *υ*_*j*_(**m**_*i*_) = ‖**m**_*i*_−**m**_*j*_‖_2_. The TPS model has a closed-form solution, which can be solved efficiently. Please refer to [18] for more details.

### Feature segmentation

For 3D US, we manually drew lumen contours for each slice due to severe boundary missing and weak image contrast. For 3D MR, a semi-automatic algorithm for carotid lumen segmentation using 2D C-V model [19] was implemented. We chose the parameters of C-V model as follows: *λ*_1_ = *λ*_2_ = 1, *μ* = *ν* = 0, *h* = *ϵ* = 1, Δ*t* = 0.1. Three user-defined lumen contours of CCA, ICA and ECA in distal slices were initialized and optimized with C-V model, and lumen contours were obtained sequentially from distal slices to bifurcation. We manually adjusted the contours where the algorithm failed. Because we separately segmented each vessel, the vessel category information of the contours was naturally included in the segmentation without extra operation.

## Experiments

In this section, we tested the proposed approach on our US-MR datasets with different measures (see Evaluation measures). We first compared our methods with state-of-the-arts. Then we verified several important settings of our TACICP algorithm. Finally the effect of parameters and segmentation noise were investigated. In all the experiments except Section, *w*_1_ was set to 1.

### Data acquisition

Because there is no public dataset, we collected our own US-MR datasets for evaluation. Three-dimensional US images and 3D multi-contrast MR images were acquired from 6 healthy volunteers without carotid plaque and 5 patients with carotid plaques consecutively. All the subjects were older than 58 years old. Six of them are male.

All the subjects received US scans for bilateral carotid arteries except 2 patients with one-side carotid arteries. Data were acquired perpendicularly to carotid arteries centered at bifurcation using a Philips iU22 US system (Philips Medical Systems, Bothell, WA, USA) with a VL13-5 linear array transducer, which has a center frequency of 7.5MHz. The linear transducer is driven by a motor to rotate with a virtual tilting axis at a certain point above the face of transducer. Volumetric data was acquired and reconstructed to slices in Cartesian coordinates with an approximate voxel size of 0.2 × 0.1 × 0.2*mm*^3^. The final 3D volume in DICOM format is composed of 256 2D images with the distance of 0.1mm.

Two black-blood imaging sequences including Motion-sensitized driven Equilibrium prepared Rapid Gradient Echo (MERGE) [20] and Simultaneous Noncontrast Angiography and intraPlaque hemorrhage (SNAP) [21] were used to obtain 3D multi-contrast MR carotid images on a Philips Achieva 3.0T TX MR system (Philips, Best, the Netherlands). Coronal images with a round 0.4mm slice thickness were acquired with an in-plane resolution of 0.35 × 0.35*mm*^2^ for MERGE images and with an in-plane resolution of 0.39 × 0.39*mm*^2^ for SNAP images.

**Fig 3:**
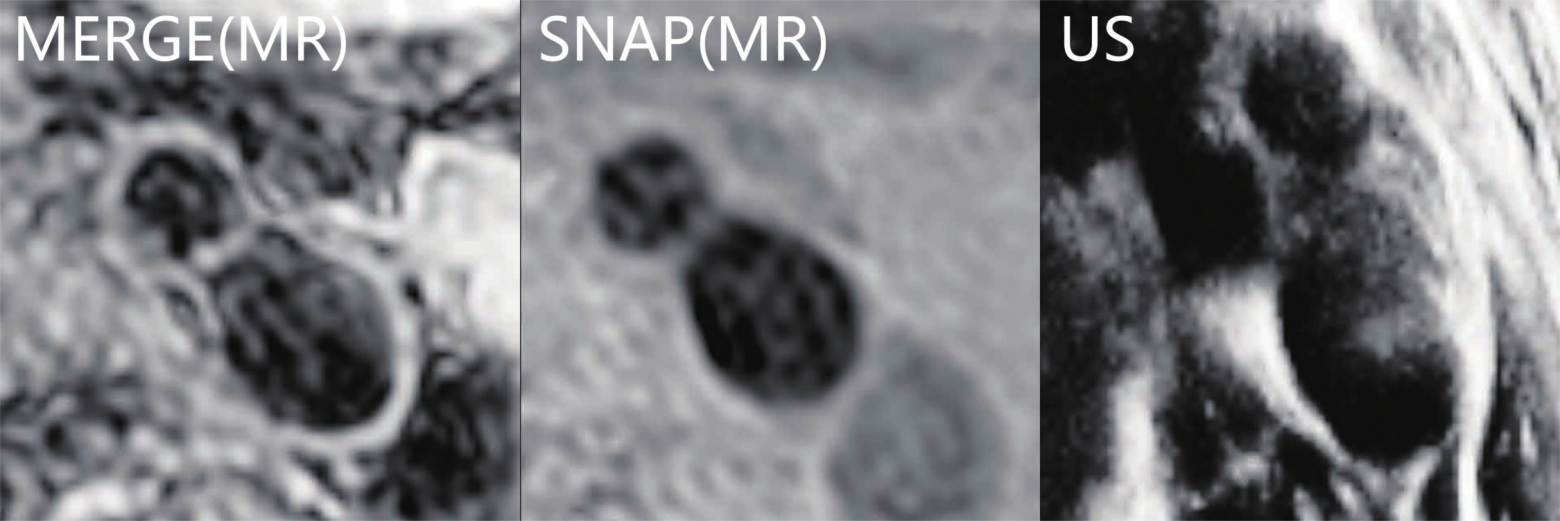
Example slices near the carotid bifurcation from a healthy volunteer for MERGE (left), SNAP (middle), and US (right) images.

All the experiments were performed on 2 dataset pairs: *US-MERGE* with 20 image sets, and *US-SNAP* with 18 image sets (two SNAP images of one healthy volunteer were discarded due to poor image quality). Examples for US and MR images were displayed in Fig 3. For point set registration, about 3500 points for surface feature and 350 points for centerline feature were used in each image.

### Evaluation measures

Manual lumen segmentations and their labels were implemented as ground truth. Two-dimensional lumen contours with labels for each slice were segmented by two experienced reviewers, where each modality was operated by one separate reviewer. These contours were reconstructed into 3D surfaces with interpolation [22] for evaluation.

The main measure for registration is based on a modified version of mean surface distance (MSD), which is a 3D-based metric calculated between the fixed surface and the transformed moving surface. The original MSD [11] uses the mean distances between the closest corresponding point pairs from two surfaces. In carotid application the matching point pairs ECA-ICA are meaningless, so we restrict the corresponding point pairs by using the labels as in the registration algorithm. We call the new metric *Labeled Mean Surface Distance (LMSD)*:

$$\text{LMSD}=\frac{1}{n}\sum_{i=1}^{n}\min_{q\in S_{f}\left(L_{p_{i}}\right)}{∥p_{i}-q∥}_{2}$$ (10)

where **p**_**i**_ is a point on the registered moving surface, **q** is a point on the subset of fixed lumen surface *S*_*f*_(*L*_**p**_*i*__) with all the points allowed to match the label of **p**_**i**_, and *n* is the total number of points on the moving surface. Because the label of the vessels near bifurcation may be uncertain, CCA-ICA and CCA-ECA pairs are allowed for evaluation. LMSD is also employed in automatic labeling strategy as a measure of label configurations.

The LMSD can only reflect the global accuracy for registration. To evaluate the local accuracy, we also define labeled maximum surface distance (LMAXD) as the maximum distance among all the closest corresponding point pairs:

$$\text{LMAXD}=\max_{i=1,\cdots ,n}\min_{q\in S_{f}\left(L_{p_{i}}\right)}{∥p_{i}-q∥}_{2}$$ (11)

The LMSD or LMAXD alone is obscure to assess the registration robustness in application. To evaluate the success rate of registration algorithm for the whole dataset, we introduce a new dataset-based measure named *Registration Success Rate (RSR)* w.r.t a certain success discrimination threshold, which is defined by the ratio of cases with LMSD more than the threshold (unit: mm) in the dataset:

$${\text{RSR}}_{x}=\frac{n_{\text{LMSD}<x}}{n}$$ (12)

We use a threshold of 1.5mm as suggested in [7] for evaluation.

In the experiments, we applied significance tests on LMSD and LMAXD between paired data. When both of the data pass the Kolmogorov-Smirnov normality test, paired t-test (*p* < 0.05) is chosen. Otherwise, non-parametric Kruskal-Wallis test (*p* < 0.05) is used.

### Comparison with other feature-based algorithms

Several state-of-the-art feature-based registration methods for multi-modal carotid images were implemented to compare with our TACICP algorithm, including surface-based rigid ICP algorithm (S-ICP) [8], rigid Gaussian mixture models based point set registration with centerlines (C-GMM) [23], and thin-plate-spline robust point matching methods (TPS-RPM) [24] with centerlines (C-RPM) or surfaces (S-RPM). The latter two methods were used in [11] as a feature-based part of hybrid model method.

To make a fair comparison, same centerlines or surfaces were used as inputs in all compared methods. We set the regularization parameter *λ* = 1 as in [11]. And as in [11], the TPS-RPM methods used rigid C-GMM algorithms for initialization.

Average LMSD, LMAXD and RSR_1.5_ were evaluated for different algorithms. Significance test (*p* < 0.05) was performed between the average LMSD and LMAXD of TACICP and that of the other algorithms. The average registration time of all the algorithms was also counted in the same environment with the same dataset.

### Automatic labeling

To verify the effectiveness of automatic labeling strategy, we evaluated LMSD after rigid CICP for both candidate label configurations on US-MERGE and US-SNAP datasets. To compare fairly, the reference labels for calculating LMSD were the same with the labels in registration for each configuration. Significance test (*p* < 0.05) was performed between two configurations.

### CICP algorithm in two steps

We studied the importance of labels in CICP algorithm by comparing CICP with ICP algorithm in two step separately. Average LMSD and LMAXD were calculated on US-MERGE and US-SNAP datasets for two algorithms in the rigid and non-rigid steps. Significance test (*p* < 0.05) was performed between CICP and ICP algorithms. RSR_1.5_ was also calculated to evaluate the overall performance on the datasets. Both the CICP and ICP algorithm in non-rigid step used the same initialization by rigid CICP algorithm for a fair comparison.

### Two-step framework

To demonstrate the necessarity of both rigid and non-rigid registration steps, we compared the two-step CICP algorithms with single-step versions, i.e. only rigid initialization step or only non-rigid refinement step. For single-step CICP algorithm, we used correct configuration of labels for registration. Average LMSD, LMAXD and RSR_1.5_ were calculated and significance test (*p* < 0.05) was performed between the average LMSD and LMAXD of two-step CICP and that of single-step versions.

### Effect of conditional distance weights

In TACICP algorithm, the conditional distance weight on the misalignment between CCA-ICA pair or CCA-ECA pair (i.e. *w*_1_) is the only parameter. Different values were tested from 1.0 to 5.0 with an interval of 0.5. Infinite weight was also involved for comparison. In practical, *w*_1_ was set to 10000 to represent the infinite weight.

### Effect of segmentation errors

The accuracy of feature-based registration algorithm relies on the accuracy of manual segmentations. Independent Gaussian noise with zero mean were added to x and y coordinate in each 2D contour to investigate the dependency on segmentation error of contours. We tested different amplitude (standard deviation) of Gaussian noise, from 0.2 to 2.0 mm with a step of 0.2mm. For each standard deviation, we repeatedly added perturbation on the original contours and ran TACICP algorithm 3 times. The average increment of LMSD after perturbation, notated ΔLMSD, was calculated on both datasets.

### Registration errors of different positions

We compared the distribution of registration errors on different positions between our algorithm and single step CICP. Average LMSD was calculated for each slice and aligned by the relative distance from the MR bifurcation slices (the closest slice to vessel bifurcation). We chosen the distance interval as [-10mm, 8mm] due to the image size of US.

### Implementation details

We developed an in-house registration software Medical Image Registration, Visualization and Analysis Platform (MIRVAP) for registration with the ICP and TACICP. The MIRVAP software was developed in Python based on VTK [25] and ITK [26].

Open source software GMMREG [23] was used to implement the state-of-the-art feature-based methods GMM and TPS-RPM. Interfaces between the softwares were also handled by the MIRVAP.

## Results

All the experiments were performed in the MIRVAP. They were run on 64-bit Windows 8 OS with an Intel i7-4700HQ CPU and 8 GB of RAM.

### Comparison with other feature-based algorithms

**Fig 4:**
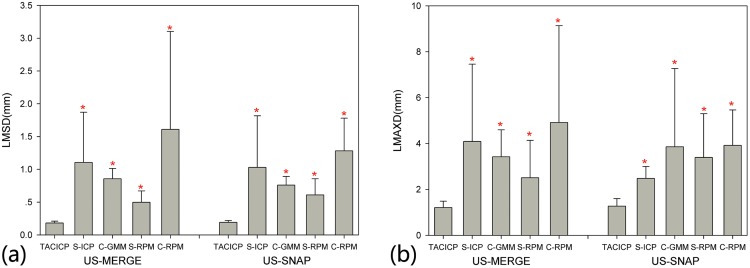
Comparison of different feature-based algorithms using average LMSD (a) and LMAXD (b) on US-MERGE and US-SNAP datasets. An asterisk indicates statistically significant (*p* < 0.05) reduction in average LMSD or LMAXD as to TACICP algorithm.

Fig 4 showed the results for comparison with the state-of-the-art feature-based algorithms. From Fig 4, our TACICP algorithm significantly outperformed all the other algorithms on both dataset evaluated by LMSD and LMAXD, which produced an average LMSD of 0.18 ± 0.03mm (US-MERGE) or 0.19 ± 0.03mm (US-SNAP) and an average LMAX of 1.22mm (US-MERGE) or 1.28mm (US-SNAP). It reached 100% RSR with level of 1.5mm on both datasets. And the computation time for proposed algorithm (within 2 minutes) was shorter than S-RPM using the same surface feature and same TPS model (see Table 1). Though rigid methods and C-RPM were faster, their registration performance was much poorer.

**Table 1:** RSR_1.5_ and computation time with the same configuration for different feature-based algorithms on US-MERGE and US-SNAP datasets.

Metric	Dataset	TACICP	S-ICP	C-GMM	S-RPM	C-RPM
RSR_1.5_/%	US-MERGE	**100.0**	80.0	**100.0**	**100.0**	80.0
	US-SNAP	**100.0**	88.9	**100.0**	**100.0**	83.3
Time/s	US-MERGE	107.94	**0.23**	1.58	2436.24	20.89
	US-SNAP	113.51	**0.20**	0.93	2919.81	15.55

**Fig 5:**
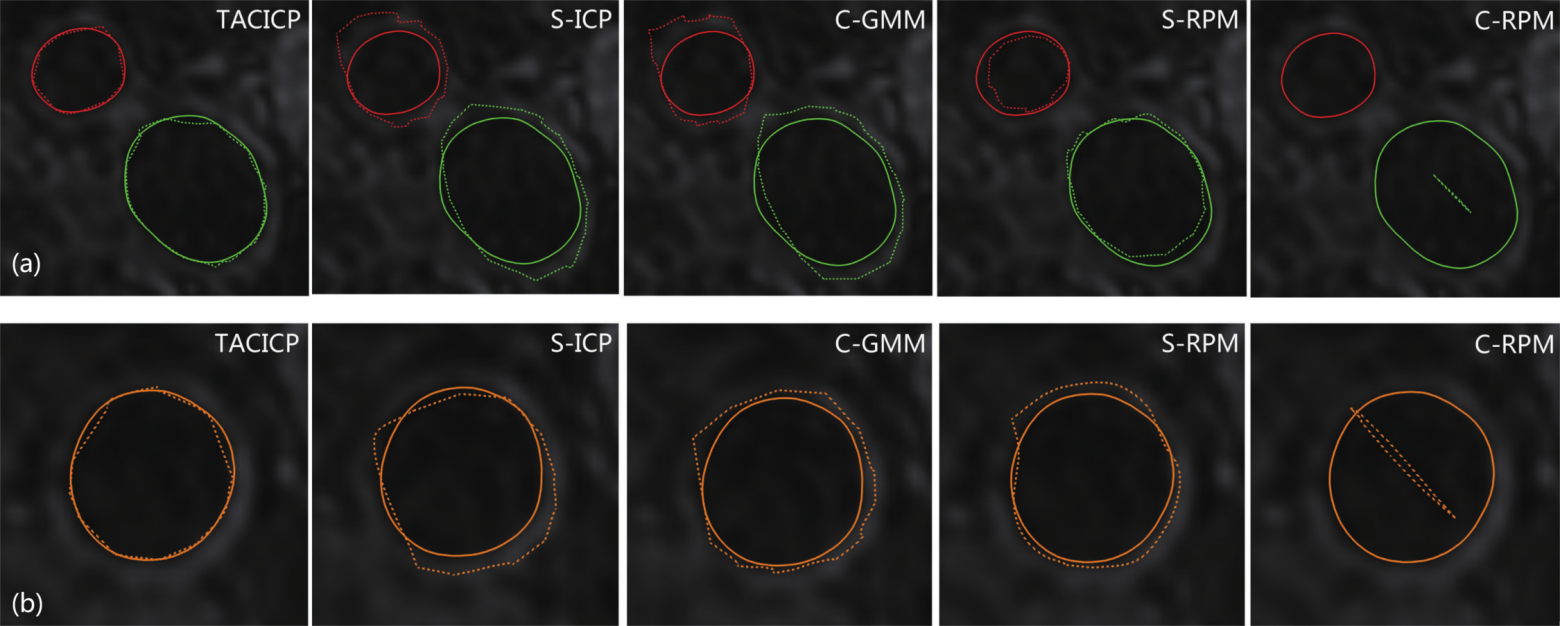
Lumen contours of CCA (orange), ICA (green) and ECA (red) in single MR (MERGE) slice with ICA and ECA (a) or CCA (b) using different feature-based algorithms. The solid lines represent the MR contours, and the dash lines represent the transformed US contours drew on the same slice.

Fig 5 showed the contours of both MRI and US simultaneously. All the non-rigid algorithms achieved better results than rigid methods except C-RPM algorithm, which failed to align the contours with only centerline input. This is because with only centerline features, C-RPM encountered over-fitting for registration in the neighborhood of centerlines due to the flexibility and locality of TPS model. Among all the results, the contours of MR and US matched the best after registration using our TACICP algorithm.

### Automatic labeling

**Fig 6:**
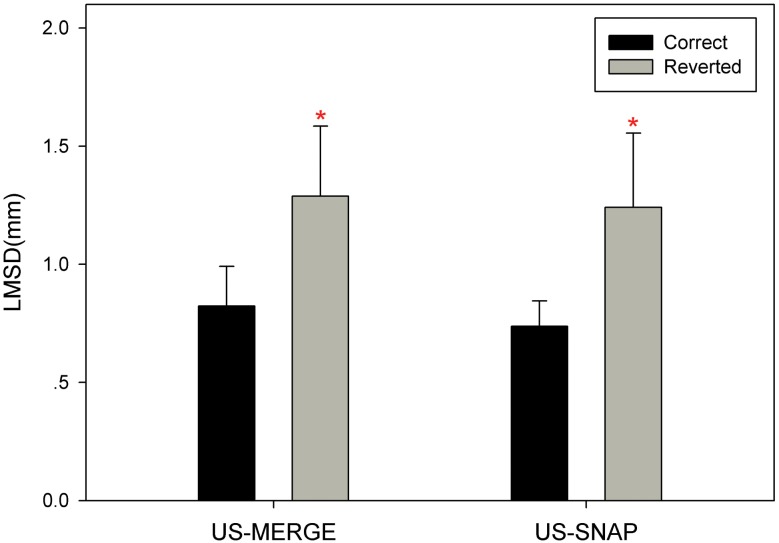
Comparison of correct label configuration (dark) and reverted configuration (light) with average LMSD on US-MERGE and US-SNAP datasets. An asterisk indicates statistically significant (*p* < 0.05) increment in average LMSD from correct configuration to reverted one.

Fig 6 showed the LMSD for each configuration. The correct configuration represents the one consistent with ground truth labels from reviewers, and the reverted configuration represents the one oppose to ground truth. LMSD of reverted configuration after rigid registration was significantly larger than that of correct configuration. Moreover, the minimum difference of LMSD between correct configuration and reverted configuration among all cases is 0.14mm in US-MERGE dataset and 0.21mm in US-SNAP dataset. This implied that by LMSD the correct configuration can be easily distinguished from the wrong one.

### CICP algorithm in two steps

**Fig 7:**
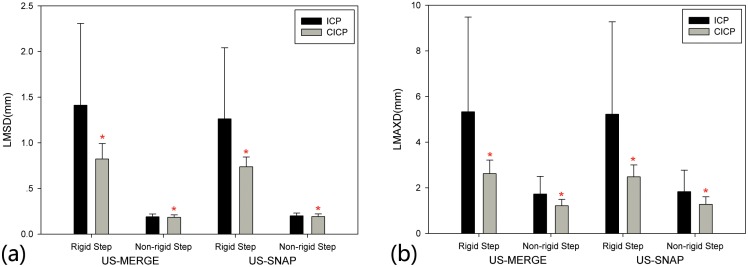
Comparison of ICP (dark) and CICP (light) in two steps with (a) average LMSD and (b) LMAXD on US-MERGE and US-SNAP datasets. An asterisk in (a) and (b) indicates statistically significant (*p* < 0.05) reduction in average LMSD or LMAXD from ICP to CICP.

Fig 7 showed the experiment results for both rigid and non-rigid registration steps. In rigid CICP, average LMSD was reduced from 1.41mm to 0.82mm and from 1.26mm to 0.74mm for the US-MERGE and US-SNAP datasets respectively with statistically significant difference compared with ICP algorithm. And RSR at 1.5mm level was increased to 100% on both datasets(see Table 2), shown the robustness of CICP algorithm in rigid initialization step. The LMAXD showed the consistent improvement. For non-rigid step, labels significantly increased the accuracy of CICP algorithm, which was reflected clearly in LMAXD with reduction of more than 0.5mm in average with smaller variance. With to LMSD, the reduction was relatively small but significant due to improvement on each image pair in registration accuracy.

**Table 2:** RSR_1.5_ of ICP and CICP algorithms in two steps on US-MERGE and US-SNAP datasets.

Metric	Dataset	Rigid Step		Non-rigid Step	
		ICP	CICP	ICP	CICP
RSR_1.5_/%	US-MERGE	65.0	**100.0**	**100.0**	**100.0**
	US-SNAP	66.7	**100.0**	**100.0**	**100.0**

### Two-step framework

**Fig 8:**
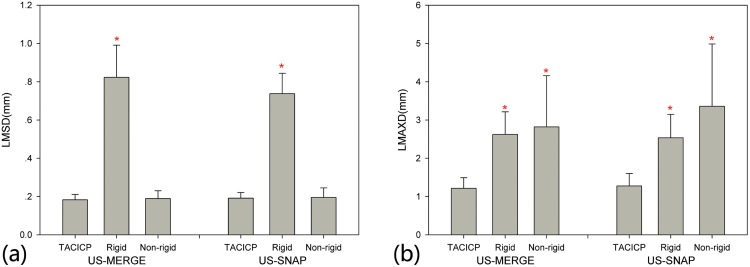
Comparison of CICP with different steps using (a) average LMSD and (b) LMAXD on US-MERGE and US-SNAP datasets. An asterisk in (a) and (b) indicates statistically significant (*p* < 0.05) reduction in average LMSD or LMAXD as to TACICP algorithm.

Fig 8 showed the registration results with different step combinations on LMSD and LMAXD. With the same contour information, non-rigid model reached superior accuracy compared with rigid model, whose LMSD was reduced by approximate 75%. Though LMSD of two-step CICP algorithm was only slightly better than single-step non-rigid CICP algorithm, LMAXD was significantly reduced by more than one half in Fig 8(b). And LMAXD of single-step non-rigid method was even larger than rigid CICP algorithm.

**Fig 9:**
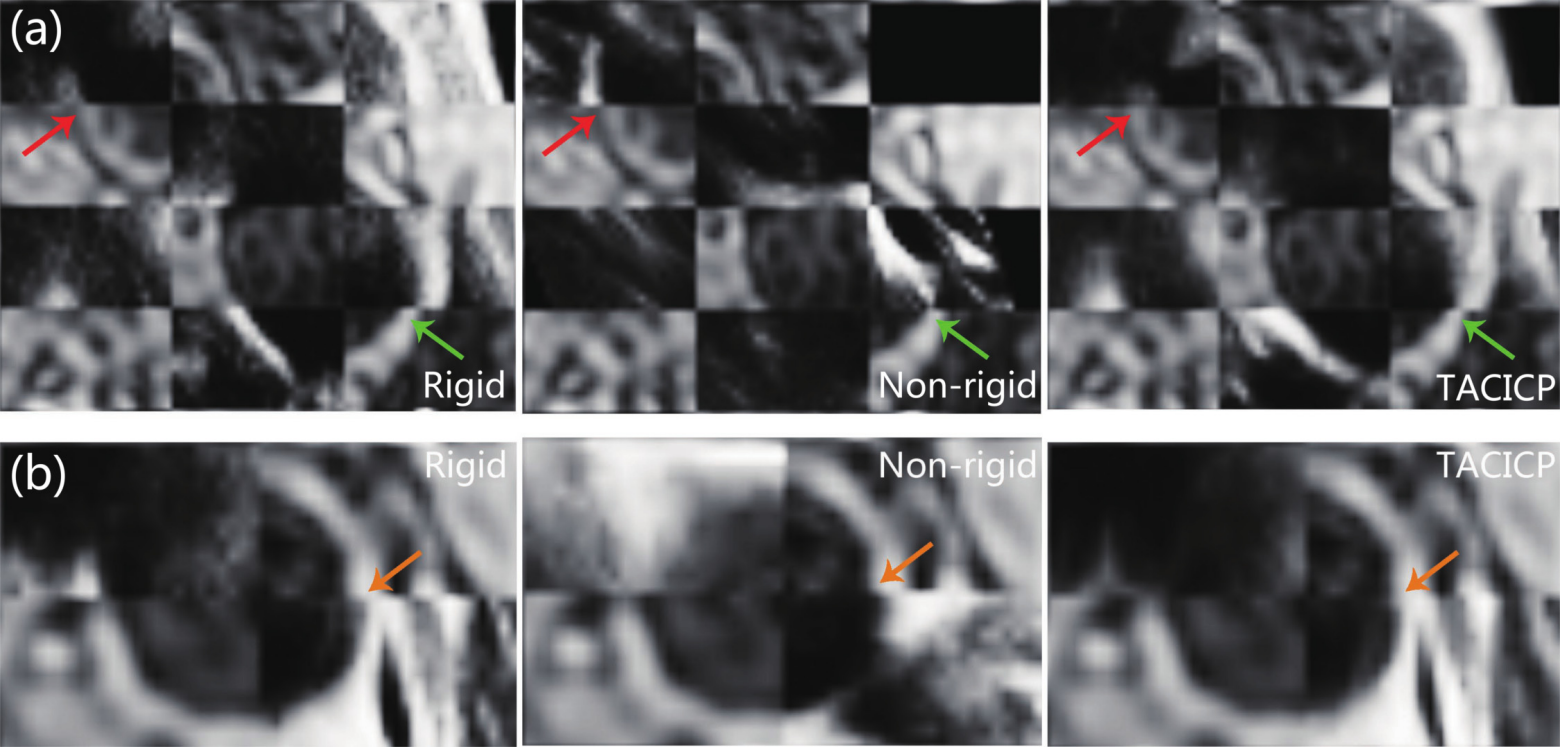
Checkerboard views of single slice with ICA and ECA (a) or CCA (b) for MR (MERGE) and US images using CICP algorithm with different step combinations. The patches in top left are from US images. US patches and MR patches appear alternately in the checkerboards. The arrows show the boundary between MR carotid lumen and US carotid lumen from CCA (orange), ICA (green) and ECA (red).

A representative result of two steps was shown in Fig 9 with checkerboard views. US patches and MR patches appeared alternately in the checkerboards. The MR carotid lumen boundary and the US boundary matched better using two-step CICP algorithm indicated with arrows.

### Effect of conditional distance weights

**Fig 10:**
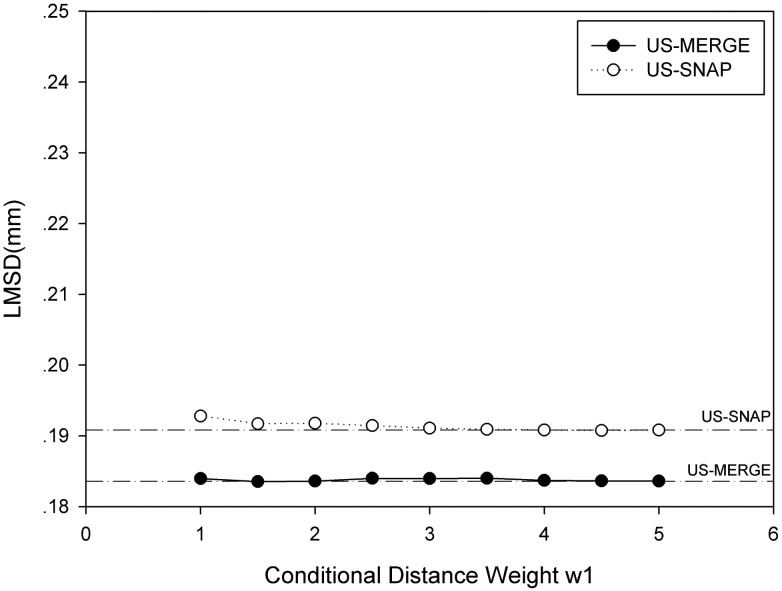
Average LMSD with different conditional distance weight of CCA-ECA or CCA-ICA pair on US-MERGE (dark) and US-SNAP (light) datasets. The dash line indicated the average LMSD with approximately infinite weight (*w*_1_ = 10000) on both datasets.

From Fig 10, registration error was robust to different penalty weights from one to infinite on both US-MERGE and US-SNAP dataset. The overall change in LMSD was less than 0.01mm in all the weights included the infinite weights. The possible reason is that the proportion of points near the bifurcation is relatively small, which means that the matches of CCA-ECA or CCA-ICA are rare. So the change in weights makes less impact on the final registration results.

There are another interesting results in this experiment. In theory, a weight of 1 or infinity is the sufficient condition of the convergence for CICP algorithm (See the proof in the Appendix). But according to the results, for all the weights CICP converged within at most 3 iterations in the rigid step, and at most 2 iterations in the non-rigid step. This may because the condition Eq (20) was rare to be violated. So the inequality Eq (18) will be satisfied almost all the time.

### Effect of segmentation errors

**Fig 11:**
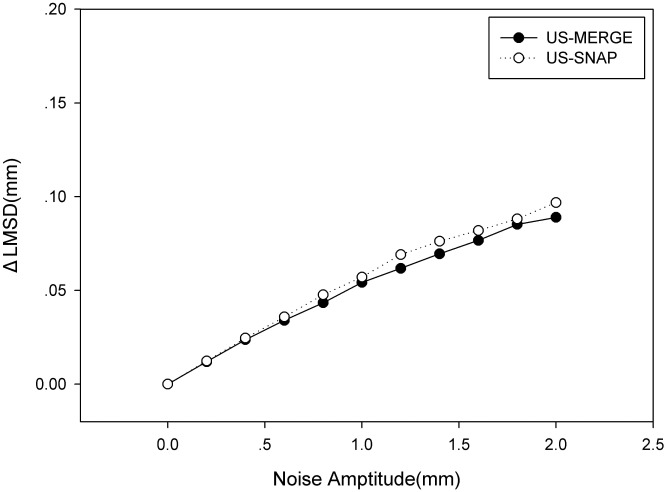
Average ΔLMSD using TACICP algorithm with different amplitude of zero mean Gaussian noise on the contours on US-MERGE and US-SNAP datasets.

The registration error of TACICP increased as the amplitude of noise on the contours increased from Fig 11. But we found that the change of LMSD was relatively small. Even with noise of 2mm (larger than average thickness of carotid lumen), the increment of LMSD was less than 0.1mm in both datasets. So TACICP algorithm was robust to the segmentation error.

### Registration errors of different positions

**Fig 12:**
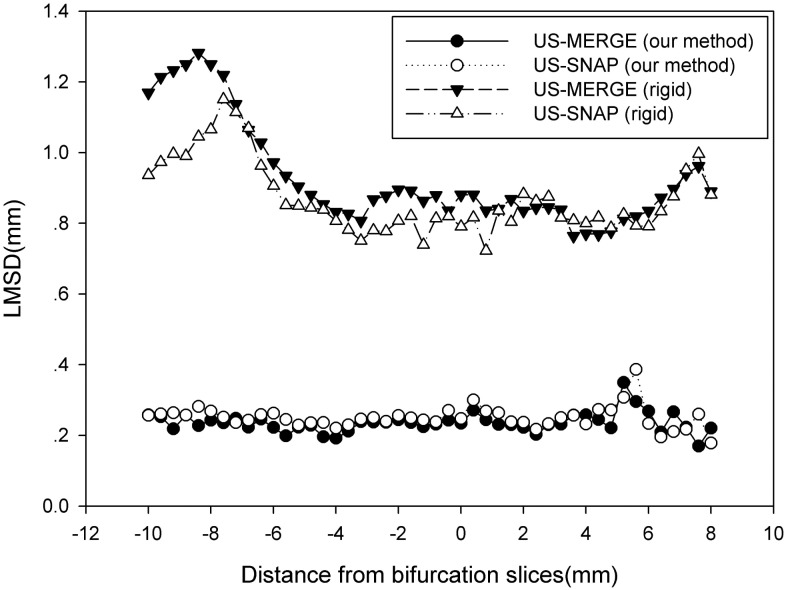
Average LMSD using TACICP algorithm and rigid CICP algorithm for different relative distance from MR bifurcation slices (the closest slice to vessel bifurcation) on US-MERGE and US-SNAP datasets.

Fig 12 showed the LMSD in each positions as a function of the distance from the bifurcation to that plane for TACICP algorithm and rigid CICP algorithm. The registration errors were below 0.4mm in all the positions, which were significantly smaller than that of single step algorithm.

## Discussion

### Two-step registration framework

In rigid transform initialization, an automatic labeling CICP algorithm was designed using centerline feature. Centerline represents the mean position set of surface points for each slice, which can be viewed as the output of a generalized mean filter applied on the surface to reduce the influence of the contour noise. Moreover, using centerline instead of surface can reduce the computation cost due to fewer points. However, only centerline can not provide enough information for rigid registration because it discards the diameter information of vessels. A 180° mis-registration may occur between ECA and ICA when the geometry of ECA and ICA are approximately symmetric with respect to CCA. Labels constrain the freedom for rigid registration, therefore increase its robustness.

In non-rigid refinement, surface feature instead of centerline was used to calculate the optimal TPS model for better local accuracy. Introduction of labels brings a more precise matched point set for transform stage, which is important for non-rigid registration accuracy due to its flexibility compared with rigid model. As results, our algorithm produced the best performance.

The coarse-to-fine strategy is critical for robustness and accuracy of our method. Only rigid registration can not handle the practical condition because of the different patient positions during US and MR scans. So the registration errors of only rigid step were higher in our experiments compared with that of non-rigid model.

In the meanwhile, CICP with only non-rigid step may converge to local minimum. Because the fitting accuracy of TPS model is strongly dependent on the selection of matching points, a bad initial position will generate bad matching results and then produce a bad registration transformation, which tends to imbalance local registration errors (a large LMAXD). By contrast, two-step CICP algorithm achieved best performance in both LMSD and LMAXD due to a good initialization. So both the rigid initialization step and the non-rigid refinement step are necessary for registration.

### Evaluation metrics for carotid image registration

To evaluate the registration algorithms, most of formal literatures used distance-based (matched points registration error [10], MSD [8][11]) or area-based (DSC [11]) measures. In the meanwhile, visual inspection was employed to estimate the registration success rate in [9].

We employed LMSD, LMAXD and RSR simultaneously for evaluation. While LMSD represents the overall registration errors of the vessels, LMAXD reveals the distribution balance of local registration errors. RSR is more objective for evaluation of registration success compared to subjective visual inspection. In our experiments, we found that single metric can not comprehensively evaluate the non-rigid registration because of the flexibility of TPS model. For instance, LMSD of single-step non-rigid CICP and two-step CICP were nearly the same, while the latter showed more accuracy results on LMAXD due to more precise local registration (see Two-step framework). So multiple evaluation measures should be employed.

### Comparison with state-of-the-art methods

We compared TACICP algorithm with other feature-based algorithms and showed promising registration accuracy. Here we also implemented some state-of-the-art non-feature-based registration methods for multi-modal images using open source software Elastix [27] with the same parameters in [11]: hybrid model method and mutual information method. Because the semi-automatic algorithms for centerline extraction in [11] did not perform well, centerlines from manual segmented contours were used for inputs.

**Fig 13:**
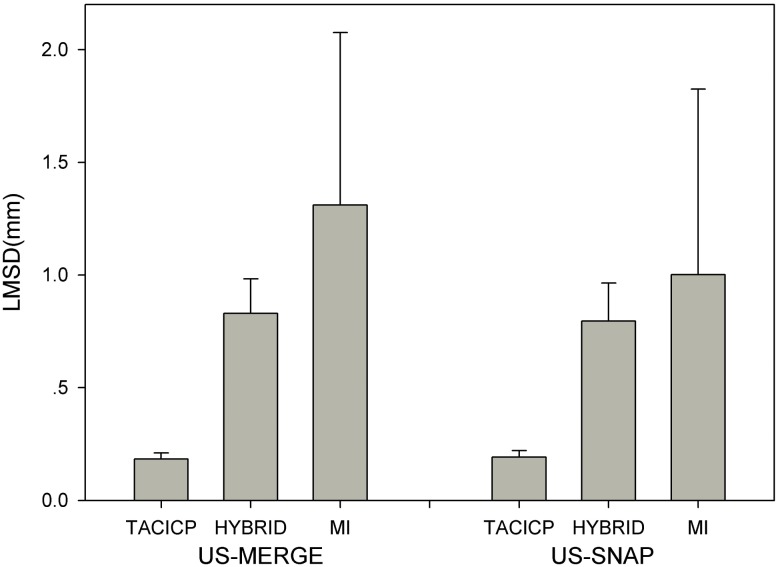
Comparison of registration results of TACICP algorithm with the state-of-the-art intensity-based and hybrid algorithms using average LMSD on US-MERGE and US-SNAP datasets. HYBRID represents hybrid model method. MI represents mutual information method.

Fig 13 shows the results. The TACICP algorithm significantly outperformed all the methods on both datasets. The comparison should be careful between our TACICP methods with other non-feature-based methods because feature-based algorithms used the information of segmentation contours that was also used for evaluation.

### Other applications for TACICP algorithm

We also tested proposed TACICP algorithm on multi-contrast (from different imaging sequences) and multi-temporal (at different time points) MR datasets for carotid imaging. Three dimensional MERGE and SNAP images (totally 18 image sets) and three extra MERGE images from patients scanned in another time point (totally 6 image sets) were registered with proposed algorithm. Table 3 showed that proposed algorithm achieved the accuracy of approximate 0.2mm with small variance in the multi-contrast and multi-temporal registration.

**Table 3:** Results for multi-contrast and multi-temporal registration using TACICP algorithm. The second column represent the registration errors evaluated with average LMSD. The last column represents LMAXD.

	LMSD/mm	LMAXD/mm
Multi-contrast	0.21 ± 0.02	1.03 ± 0.10
Multi-temporal	0.18 ± 0.04	0.94 ± 0.17

In addition to carotid image registration, the proposed TACICP algorithm can also be applied to other vascular beds with similar bifurcation structure, such as abdominal artery and femoral artery.

### Limitation

A limitation of our work is that we just used the manual segmentation results without automatic methods. The state-of-the-art centerline or contour automatic extraction methods [28][29][30] did not work on our datasets because of bad image quality of US images.

However, after evaluation our method is robust enough to handle segmentation errors that are comparable with vessel thickness, which relax the expertise requirement of segmentation for users. It may further improve registration accuracy if combination or iterating strategy is employed between segmentation and point set registration [31][32][33], which may be a promising direction for our future work.

## Conclusion

In conclusion, proposed TACICP algorithm can serve as a robust and accurate registration method for 3D carotid imaging. It obtains an average LMSD of 0.18 ± 0.03*mm* on the US-MERGE dataset and 0.19 ± 0.03*mm* on the US-SNAP dataset without any failure case at the threshold of 1.5mm, which shows the superior performance compared with the other state-of-the-art feature-based methods. Though with limitation, our two-step algorithm has potential to be a practical carotid registration method in clinical application.

## Appendix

The convergence of CICP algorithm is proved here. The definition of notation can be found in Rigid initialization step. Let $M_{k}={\left\{m_{i,k}\right\}}_{i=1}^{N_{m}}$ be the moving point set before *k*^*th*^ iteration, and $F_{k}={\left\{f_{i,k}\right\}}_{i=1}^{N_{m}}$ be their closest corresponding points in the fixed point set. So mean squared error(MSE) *e*_*k*_ is

$$e_{k}=\frac{1}{N_{m}}\sum_{i=1}^{N_{m}}{∥m_{i,k}-f_{i,k}∥}_{2}$$ (13)

After calculating and applying the optimal transformation *T*_*k*_, the MSE $e_{k}^{\prime }$ becomes

$$e_{k}^{\prime }=\frac{1}{N_{m}}\sum_{i=1}^{N_{m}}{∥m_{i,k+1}-f_{i,k}∥}_{2}$$ (14)

where **m**_*i*, *k*+1_ = *T*_*k*_(**m**_*i*, *k*_). Obviously we have $e_{k}^{\prime }\leq e_{k}$, or the identity transformation will be a better choice than *T*_*k*_.

Next, the new closest corresponding points in the fixed points $F_{k+1}$ can be obtained:

$$\begin{array}{cc} f_{i,k+1}=\underset{f_{j}\in F}{argmin}W_{L_{f_{j}},L_{m_{i,k+1}}}∥ & f_{j}-m_{i,k+1}{∥}_{2}\\ & i=1,2,\cdots ,N_{m} \end{array}$$ (15)

From Eq (15), it is clear that

$$\begin{array}{cc} W_{L_{f_{i,k+1}},L_{m_{i,k+1}}}{∥f_{i,k+1}-m_{i,k+1}∥}_{2} & \\ \leq W_{L_{f_{i,k}},L_{m_{i,k}}}{∥f_{i,k}-m_{i,k}∥}_{2} & \\ i=1,2,\cdots & ,N_{m} \end{array}$$ (16)

For all the selected corresponding points in the fixed point set, the weights must be finite. To simplify the notation, let *a*_*i*, *k*_ = *W*_*L*_**f**_*i*, *k*__, *L*_**m**_*i*, *k*___. So Eq (16) can rewrite as

$$\begin{array}{cc} a_{i,k+1}∥f_{i,k+1}-m_{i,k+1}{∥}_{2}\leq a_{i,k}{∥f_{i,k}-m_{i,k}∥}_{2} & \\ i=1,2,\cdots & ,N_{m} \end{array}$$ (17)

To guarantee the convergence of CICP, we should have

$$∥f_{i,k+1}-m_{i,k+1}{∥}_{2}\leq {∥f_{i,k}-m_{i,k}∥}_{2}$$ (18)

so that the following inequality is satisfied:

$$0\leq e_{k+1}^{\prime }\leq e_{k+1}\leq e_{k}^{\prime }\leq e_{k}\;\text{forall}k$$ (19)

So from Eq (17), we have

$$a_{i,k}\leq a_{i,k+1}$$ (20)

for all the *i*. Because *a*_*i*, *k*_ ∈ {*w*_0_, *w*_1_}, *w*_1_ must satisfy that *w*_1_ ≤ *w*_0_, which means that *w*_1_ = 1. Note that when *w*_1_ = ∞, CICP will also converge. So *w*_1_ = 0 or *w*_1_ = ∞ is the sufficient condition for convergence.
